# Developmental activities of elite junior hockey players: an analysis of early sport specialization

**DOI:** 10.3389/fspor.2023.1253007

**Published:** 2023-10-31

**Authors:** W. J. Garland, K. L. Smith, J. C. Dixon, S. Horton

**Affiliations:** Department of Kinesiology, University of Windsor, Windsor, ON, Canada

**Keywords:** early specialization, athlete development, deliberate practice, deliberate play, long-term athlete development, sport commitment, junior hockey

## Abstract

Early sport specialization is a popular and contentious topic in the scientific literature and popular media. The lure of extrinsic rewards has led to increasing rates of specialization among young athletes, while expert recommendations promote multisport participation. The purpose of this study was to describe and analyze developmental activities of a group of elite junior hockey players in Canada. Within this context, elements of specialization were investigated in accordance with existing theoretical frameworks and long-term athlete development models to enhance the literature. Fifteen participants from the Ontario Hockey League completed quantitative retrospective interviews, detailing past sport and recreational activities. Thirty-one developmental milestones were assessed. Accumulated hours of activity were categorized in accordance with Côté's (1999) Developmental Model of Sports Participation, along with the number and types of sports in which they participated during childhood. Jayanthi et al.'s (2015) continuum was utilized to determine the age at which the athletes became moderately and highly specialized. Accrued hours of deliberate practice reported by participants increased from ages 6 to 16 years, as did competition in organized hockey games. Reported hours of deliberate play peaked at 9 years of age and decreased thereafter. Participants played a combined 16 sports other than hockey, ranging from an average of 2.0 at age 6, to a maximum average of 5.6 at 12 years old, and decreasing each year to 2.3 by age 15. The greatest number of hours in other sports was accumulated at 12 years of age. Using a three-point scale, participants considered themselves “highly specialized” at 14 years old; however, other quantitative indicators suggested this may have occurred at 12 years of age. Relative to previous research on early sport specialization, participants in this study spent more time practicing hockey, while ceasing hockey-specific play and other sports at younger ages. Despite a diverse sport history, hockey competition was initiated earlier than recommended, showing high levels of sport commitment as young as 9 years old. The early specialization path remains a popular trajectory among coaches, parents, and athletes in Canadian ice hockey.

## 1. Introduction

*Sport specialization* generally refers to concentrated training in one sport for a significant portion of the year at the expense of participation in other sport and activities (1–4); although operational definitions vary in the published literature [see (5) for further discussion]. This developmental trajectory in youth sport has become both a popular and contentious topic in the literature and popular media, as experts debate the benefits and consequences of intensified participation for young athletes (6–9). The belief that intense participation is the best route to an athletic scholarship (10), or a professional contract (11) has led to a dramatic rise in privatized programs and year-round participation, and a decrease in the number of multi-sport athletes (12–15). Yet, athletes, parents, coaches, and administrators alike, may be overlooking the documented physical and psychological consequences of this path as they pursue the external rewards associated with athletic success (4, 16).

Early sport specialization is not a new approach with respect to talent development, dating back to the 1950s when Eastern European countries introduced intensive training programs in the quest for political power through Olympic success (17, 18). Coaching philosophies gradually migrated West with promotion of year-round participation in a single sport at a young age, privatized training (19), and commercialization of sport programs (12)^1^. Modern day athletes have many motives to excel in their given domains, and the belief that intense, year-round participation is the best route to excellence has flourished (15, 28). For instance, the National Collegiate Athletic Association (NCAA) has studied the trend of early specialization and reported that more than 50% of male soccer and hockey players have specialized by 12 years of age (29), which is notably short of the completion of adolescence wherein young athletes undergo a significant period of physical maturation and might be at increased risk of injury (19, 30). Despite the many proponents of early sport diversification [e.g., (13, 31)], trends show a decrease in the number of multi-sport athletes, presumably due to private entities promoting specialization as a path to elite status [(32); see (2) for further discussion].

The popularity of sport specialization is also tied to the work of Ericsson et al. (33), whose seminal paper outlined a monotonic relationship between practice and performance, such that the more one practices, the better one will perform. This paper further supported the 10-year model proposed by Simon and Chase (34) following an examination of chess players, suggesting that 10 years of meaningful, purposeful practice is needed to demonstrate expertise in a given domain. This form of training is often referred to as *deliberate practice* and consists of highly structured, effortful activities aimed at improving specific aspects of performance (33). Support for this concept has been garnered across several domains including sport [e.g., (35–37)], although a notable distinction has accompanied sport-specific findings. Helsen and colleagues (36) reported that practice is often seen as enjoyable and highly relevant to athletes, contradicting Ericsson et al.'s (33) observations of the talented musicians who reported practice as relatively less enjoyable in comparison to other activities. Deliberate practice has reliably differentiated skill level across sport-specific studies [see (38, 39)], although not all individuals benefit equally from the effects of training (40) and early specialization is not necessarily a requirement to reach elite status (31).

In contrast to the premises put forth by the deliberate practice framework, Côté (41) explained an athlete's development through the *Developmental Model of Sport Participation* (DMSP). The first stage, referred to as “sampling” (i.e., ages 6–13), occurs when athletes experience a variety of different sports with an emphasis on fun and enjoyment. During the “specialization” stage (i.e., ages 13–15), athletes commit to one or two sports and achievement becomes more important. Finally, during the “investment” stage, athletes (over 15 years of age) commit to one sport with the hope of attaining an elite level of performance. There is a possibility of a fourth stage if a high degree of accomplishment has been attained; this is referred to as the “performance” or “perfection” stage. During this time, athletes maintain their elite status and work towards perfecting the skills of their domain.

As an alternative to the DMSP model, Balyi and colleagues (42) proposed a seven-stage model based on developmental stages, aimed at optimizing long-term athlete development (LTAD) and promoting physical activity in the general population. The first stages of “active start”, “fundamentals”, and “learn to train” promote the skills required for physical literacy and are recommended to be completed before the onset of adolescence. High performance athletes may then progress through the stages of “train to train”, “train to compete”, and “train to win” by showing key psychosocial and physical indicators. As expected with high performance, tiered systems and increasingly difficult challenges cause athletes to be filtered out at each stage, allowing fewer to move on to the subsequent stage or tier. Ideally, as these athletes are filtered out of the high-performance system, they enter the “active for life” stage with the goal of having them stay active and healthy. This seven-stage model has been adapted and implemented at the developmental level by various sport governing bodies. For instance, Hockey Canada (43) made the stages specific to the contextual and logistical constraints of minor hockey in Canada. The program is designed to increase physical literacy and life-long engagement in hockey activities by encouraging young players to participate in other activities until the age of 12, when it is recommended to increase the specificity of training towards hockey.

The cultural shift toward early sport specialization has also prompted several organizations to release position statements advising against this potentially harmful trend. For instance, the International Olympic Committee has outlined recommendations for managing the sport-life balance of young athletes in their consensus statement (44), and the American Orthopaedic Society for Sports Medicine called for improved messaging concerning specialization, due to associations with adverse outcomes for athletes (45). While a positive correlation does exist between practice and performance, the recommended amount and type of training differs at various stages of development (23, 35, 36, 46, 47). Moreover, accumulating more practice by age 15 and selection to elite junior teams does not necessarily predict success at higher levels [see (31)]. Thus, early specialization could be associated with an athlete reaching their highest level of play prematurely (48, 49).

Further, intense training without adequate rest has been identified as an independent risk factor for injury (14, 50). This risk may be attributed to repetitive movements, strength and flexibility imbalances, inadequate neuromuscular skills to prevent injury, and specialized athletes pushing themselves with more intensity (10, 14, 51). For example, a common overuse injury sustained by ice hockey players is femoroacetabular impingement (FAI),^2^ which occurs due to the repetitive biomechanics of ice skating (53). Risk factors for FAI include on-ice exposure and younger start ages, and the prevalence increases with age showing that hockey-specific movements may lead to the development of FAI (53). This is noteworthy, as hockey reports a high incidence of early specialization (54). Psychosocially, early specialization may alter relationships, create socially maladaptive behaviour, increase the risk of anxiety, depression, and burnout, and decrease intrinsic motivation (2, 4). These negative outcomes may be associated with the pressure created by overly structured, adult-driven training schedules (2, 10, 26, 55).

Following recommendations for sport diversification and specialization within the Canadian developmental system for ice hockey has had its challenges. The Canadian Hockey League (CHL) serves as the primary developmental league for the National Hockey League (56, 57), and consists of the Western Hockey League (WHL), Ontario Hockey League (OHL), and Quebec Major Junior Hockey League (QMJHL). Players may enter the CHL as early as 15 years old (58) and thus, may feel pressure to accumulate practice hours in ice hockey at the expense of other sports to demonstrate superior ability prior to that age (2). The adapted Balyi et al. (42) developmental model recommended by Hockey Canada (43) suggests hockey-specific training should be initiated at the age of 12, leaving a limited window of opportunity for players to elevate their skills above the competition. The existing developmental literature involving CHL players [e.g., (25, 59)] was published after the 1999 Molson Open Ice Summit on player development. As a consequence of this summit, Hockey Canada changed its grassroots programs, and recommended the implementation of “skills academies” throughout the country (60). These changes led to as many as 5,000 elementary and secondary schools offering hockey as part of their curricula, allowing minor hockey players as young as 12 years old to focus on activities specific to hockey (60, 61).

While empirical research regarding specialization and LTAD is found for several sports, including soccer [e.g., (23)], volleyball (24), and athletics (50)—relatively little is available for hockey. Thus, the purpose of this study was to describe and analyze the developmental activities of a group of elite junior (OHL) hockey players to investigate elements of specialization and enhance the LTAD literature for one of Canada's national sports. The developmental activities of CHL players potentially affected by changes occurring after the Open Ice Summit were considered. Accumulated hours of activity were categorized in accordance with the DMSP, along with the number and types of sports in which participants engaged during childhood. Jayanthi et al.'s (62) continuum was utilized to determine the age at which the athletes became moderately and highly specialized. Given current trends and the popularity of ice hockey in Canada, it was hypothesized that “specialization” and “investment” would occur earlier than suggested by the DMSP, and that the athletes would be considered “highly specialized” prior to recommended ages, according to Jayanthi et al.'s (62) continuum.

## 2. Methods

### 2.1. Participants

Potential participants were current or former OHL players who were born no earlier than January 1st, 1994. These inclusion criteria created a sample of elite junior hockey players who had initiated their developmental activities after the Open Ice Summit on player development (60). All participants were male and had spent a portion of their developmental years in the Kent or Essex counties of Ontario, Canada. Participants were from a relatively small geographical area in Ontario giving similar proximity to training resources. Patton (63) refers to this type of criterion-based sampling as purposeful sampling, which is intended to create an information-rich and homogeneous sample for an in-depth analysis of cases that have been determined to be important.

### 2.2. Questionnaire/instruments

The retrospective semi-structured interview script^3^ used in this study was an adapted version of the guide developed by Côté et al. (64) to obtain quantitative information regarding the developmental activities of participants in various stages throughout their childhood up to their time in the OHL. Versions of this interview have been used in several previous studies for other sports, including triathlon [e.g., (65)], volleyball [e.g., (66)], soccer [e.g., (67)], rugby [e.g., (68)] and swimming [e.g., (69)]. The information collected from the interviews was based on questions from three sections: (a) “early activities”, (b) “maturation and performance in main sport”, and (c) “relevant practice activities in main sport”. This information helped to generate longitudinal, methodical, and comprehensive histories of the participants' developmental activities. The interview script also involved closed-ended questions to add context by rating each activity on several subjective measures, such as effort, mental concentration, and enjoyment.

The “early activities” section created a list of activities that participants engaged in throughout their development. Participants were asked to recall the number of hours per week, and the number of months per year, for every year that they took part in each activity. One adaptation from Côté et al.'s (64) interview guide was a designation of peak and off-peak months. This was included to account for the changes in training hours between seasons. The “maturation and performance in main sport” section asked each participant for specific ages of developmental milestones, information regarding injuries, subjective feelings towards training intensity and resources, and personal and team accomplishments. Finally, the “relevant practice activities in main sport” section regrouped the information previously collected into periods of similar training quality and quantity. These periods helped to quantify the time and effort required for the activities identified in previous sections of the interview. All sections of the interview focused on the participants' relevant activities from the age of 6 years and throughout their development to include those pursued at the time of the interview.

To identify the age at which participants became moderately and highly specialized, the scale suggested by Jayanthi et al. (62) was utilized. Participants were asked when they chose hockey as their main sport, when they engaged in hockey for more than eight months per year, and when they first dropped out of other activities to focus their attention on hockey^4^. Participants were also asked if they had ever been told that they were not permitted to play other sports. Those who answered in the affirmative were also asked who made the request, and what rationale was provided, to gain an understanding of why participants specialized in hockey. Participants were then asked if they had ever attended a hockey academy during their development and if they had ever relocated to play hockey. This was to determine the rationale for the relocation, and if there were any trends among the participants.

### 2.3. Procedure

Current and former OHL players were approached by the first author to explain the study and its objectives. Interviews were conducted on an individual basis at a private location convenient to the participant, and each interview took roughly two hours to complete. Confidentiality was maintained by using pseudonyms for each participant. The first author assisted the participants by helping them during the interview which aided them in maintaining focus and ensured the validity and reliability of their data. These procedures received clearance from the Research Ethics Board at the authors' home institution and all participants provided their informed consent.

### 2.4. Reliability

Côté et al. (64) first proposed their interview guide to reduce the limitations of athletes’ recall accuracy. The interview procedure describes how to collect verifiable information, trace longitudinal changes in developmental activities, and assess the validity and reliability of the information given. Questions focus on the objective recall of specific episodic experiences (e.g., training hours), which are considered reliable, in part, because of the large impact that they have on athletes' lives. Côté et al. (64) found the test re-test reliability to be between .70 and .84 for these questions, and the convergent validity between athletes' recall and that of others with knowledge of their training quantity was found to be statistically reliable (e.g., parents, coaches, training partners). During subsequent studies, the test-retest reliability was determined to be between .83 and .98 (66, 70, 71). Convergent validity involving similar methods in other studies was between .76 and 1.00 (70, 72, 73). In this study, the first author had general knowledge of the developmental histories of each participant, which facilitated accurate recall of specific events, in comparison to a researcher unfamiliar with the athletes' past activities (64).

### 2.5. Data analyses

The interview systematically explored all activities that each participant had undertaken during development. This included a record of the number of hours per week and the number of months per year for each activity, allowing for the calculation of the total number of hours per year. As per the interview guide, these hours were then separated into “sport”, “artistic”, and “other” (e.g., watching TV) categories. Consistent with the methodology used in Soberlak and Côté (25), sporting activities were divided into “deliberate practice activities”, “deliberate play activities”, “organized games”, and “other sports”. The hours accumulated in each category were divided in accordance with the age groups suggested in the DMSP: sampling (i.e., ages 6–13), and specializing (i.e., ages 13–15). Descriptive statistics (i.e., mean, median, standard deviation, maximum and minimum hours) were calculated and graphed to identify trends, and analyzed to determine if transitions were consistent with those proposed in the DMSP. “Deliberate practice activities” were defined as any activities that had the purpose of improving the participants' performances in hockey [e.g., self-initiated practice, team practice, individual coaching; (33)]. “Deliberate play activities” were hockey-related activities that had the goal of providing enjoyment to the participants (41). “Organized games” were any organized hockey competitions, and “other sports” were sports other than hockey that were identified by participants. The findings highlight descriptive statistics derived from the interviews.

Due to the importance and relevance of sporting activities, each participant's involvement was categorized using Thorpe et al.'s (74) games classification model, similar to the methodology used in Berry et al. (72). The Thorpe et al. (74) model classifies all games into one of five categories based on the basic strategies and principles: invasion (e.g., hockey, soccer), net/wall (e.g., tennis, volleyball), field/run scoring (e.g., baseball), target (e.g., golf), and individual (e.g., athletics). Hockey qualifies as an invasion sport because the purpose is to invade the opponent's territory and score on the opponent's goal. Descriptive statistics of the accumulated hours of structured and unstructured involvement in each of these sport classifications were calculated, as well as the frequency of the various activities identified by participants.

According to the scale developed by Jayanthi et al. (62), all the participants in this study were considered highly specialized at the time of the interview. Therefore, the age at which each athlete first qualified as highly specialized was determined and reported with descriptive statistics. The remaining questions about playing other sports, relocation, and hockey academy attendance were included in the analysis.

## 3. Results

### 3.1. Participants

Fifteen participants were interviewed, all of whom were current or former OHL players. These athletes had a mean age of 19.6 years with the oldest athlete being 22 years of age, and the youngest being 16. The number of OHL games played by participants ranged from 51 to 294 (mean of 145, median of 139). Since all athletes had commenced playing in the OHL by 17 years of age, results were calculated up to and including 16 years of age for each athlete.

### 3.2. Accumulated hours of activity

The number of estimated activity hours that each athlete accumulated per year in “sport^5^”, “artistic^6^”, and “other” categories is reported in Table 1. For each age, the participants reported accumulating a greater number of total hours in the “sport” category, and attained greater mean, median, maximum and minimum values than the “artistic” and “other” categories combined. The maximum number of activity hours per year reported by participants occurred at the age of 13 for “sport” (16,338), 9 years old for “artistic” (774), and 13 years of age for “other” (3,508).

**Table 1:** Descriptive statistics of accumulated activity hours by age.

Category	Age										
	6	7	8	9	10	11	12	13	14	15	16
All sports											
Total	7,290	8,872	11,070	11,734	12,772	13,896	15,362	16,338	15,744	14,246	14,918
Mean	486.00	591.47	738.00	782.27	851.47	926.40	1,024.13	1,089.20	1,049.60	949.73	994.00
S.D.	433.96	387.87	459.80	497.77	548.09	560.07	572.19	525.61	468.55	338.97	296.00
Median	458	492	548	552	576	652	896	936	856	912	896
Max	1,592	1,592	1,620	1,756	2,156	2,156	2,284	2,200	2,084	1,454	1,838
Min	60	144	264	304	352	312	312	448	372	420	556
Artistic											
Total	12	144	678	774	646	630	666	542	606	240	100
Mean	0.80	9.60	45.20	51.60	43.07	42.00	44.40	36.13	40.00	16.00	6.67
S.D.	3.10	37.18	129.85	131.30	130.07	130.37	129.87	83.71	103.86	33.97	25.82
Median	0	0	0	0	0	0	0	0	0	0	0
Max	12	144	504	504	504	504	504	320	400	100	100
Min	0	0	0	0	0	0	0	0	0	0	0
Other											
Total	1,984	2,284	2,380	2,236	3,484	3,356	3,356	3,508	3,024	2,744	3,064
Mean	132.27	152.27	158.67	149.07	232.27	223.73	223.73	233.87	201.60	182.93	204.27
S.D.	239.43	233.36	230.50	226.26	410.70	407.51	407.51	402.53	225.85	217.14	245.65
Median	0	0	64	64	128	84	84	96	160	80	144
Max	800	800	800	800	1,600	1,600	1,600	1,600	800	800	800
Min	0	0	0	0	0	0	0	0	0	0	0

**Figure 1:**
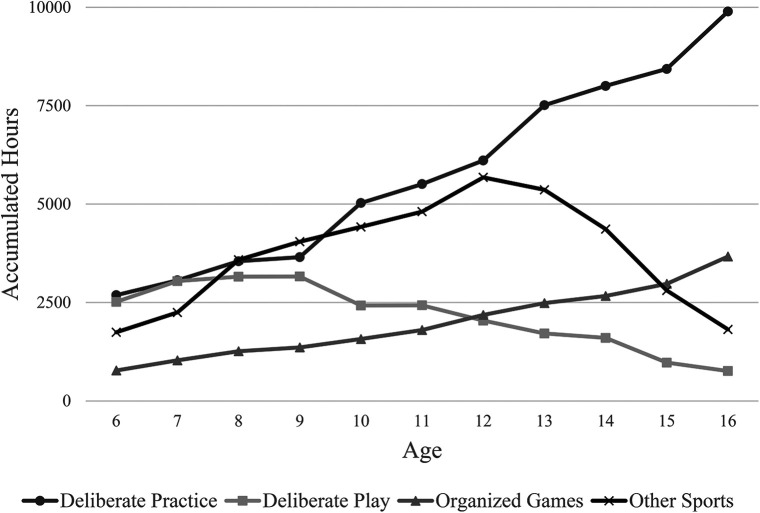
The combined accumulated hour totals for hockey-specific deliberate practice, hockey-specific deliberate play, organized hockey games and sports other than hockey at each age.

The “sport” hours accumulated were further classified as hockey-specific “deliberate practice”, hockey-specific “deliberate play”, “organized [hockey] games”, and sports other than hockey. Descriptive statistics for each of these categories are presented in Table 2, and the total accumulated hours of all participants at each age are graphed in Figure 1. The combined total hours of all participants were lowest at 6 years old (2,688) and steadily increased until participants were 16 years of age, when deliberate practice achieved its highest combined maximum (9,892), and median (568) number of hours. The highest combined total of deliberate play hours was achieved at 9 years of age (3,160), which decreased sharply thereafter. Despite the decline in combined accumulated hours of deliberate play, participants had the highest median (156) hours at 11 years of age. The number of hours that participants engaged in organized games gradually increased at each age, with the highest combined total (3,668), and median (256) at age 16. Finally, when considering sports other than hockey, participants had the highest combined total of hours (5,342), median hours (282), and individual maximum hours at 12 years of age (1,300), at which point the commitment to non-hockey related sport activities decreased steadily.

**Table 2:** Descriptive statistics for accumulated sport hour categories.

Category	Age										
	6	7	8	9	10	11	12	13	14	15	16
Hockey-specific deliberate practice											
Total	2,688	3,064	3,548	3,652	5,028	5,508	6,108	7,512	8,002	8,434	9,892
Mean	179.20	204.27	236.53	243.47	335.20	367.20	407.20	500.80	533.47	562.27	659.47
S.D.	202.89	183.92	185.95	185.69	359.71	345.81	345.37	329.37	331.02	269.93	284.76
Median	120	120	168	172	172	252	284	444	444	516	568
Max	744	744	744	744	1,452	1,452	1,452	1,452	1,452	1,088	1,344
Min	0	24	64	64	64	88	88	124	124	192	256
Hockey-specific deliberate play											
Total	2,516	3,044	3,156	3,160	2,424	2,428	2,040	1,712	1,600	972	760
Mean	167.73	202.93	210.40	210.67	161.60	161.87	136.00	114.13	106.67	64.80	50.67
S.D.	230.58	218.83	219.91	219.69	141.10	137.35	124.83	118.69	120.00	68.84	86.01
Median	80	136	124	124	124	156	96	72	72	48	8
Max	716	716	716	716	476	476	476	476	476	224	252
Min	0	8	8	8	0	0	0	0	0	0	0
Organized hockey games											
Total	774	1,030	1,262	1,358	1,572	1,800	2,184	2,486	2,666	2,974	3,668
Mean	51.60	68.67	84.13	90.53	104.80	120.00	145.60	165.73	177.73	198.27	244.53
S.D.	41.43	30.42	57.03	53.65	54.30	54.53	71.58	63.62	63.86	69.95	36.06
Median	48	72	72	72	96	96	144	160	172	198	256
Max	144	144	264	264	264	264	288	288	288	296	296
Min	0	24	24	48	48	48	48	72	72	72	168
Sports other than hockey											
Total	1,408	1,910	3,248	3,708	4,084	4,472	5,342	5,028	4,028	2,470	1,814
Mean	93.87	127.33	216.53	247.20	272.27	298.13	356.13	335.20	268.53	164.67	120.93
S.D.	77.77	99.17	226.83	256.90	249.90	260.72	286.93	266.27	211.41	120.64	85.43
Median	68	108	160	190	196	232	282	276	196	212	128
Max	296	376	948	1,108	1,108	1,172	1,300	1,080	812	332	300
Min	0	0	0	36	72	64	64	60	12	0	0

**Figure 2:**
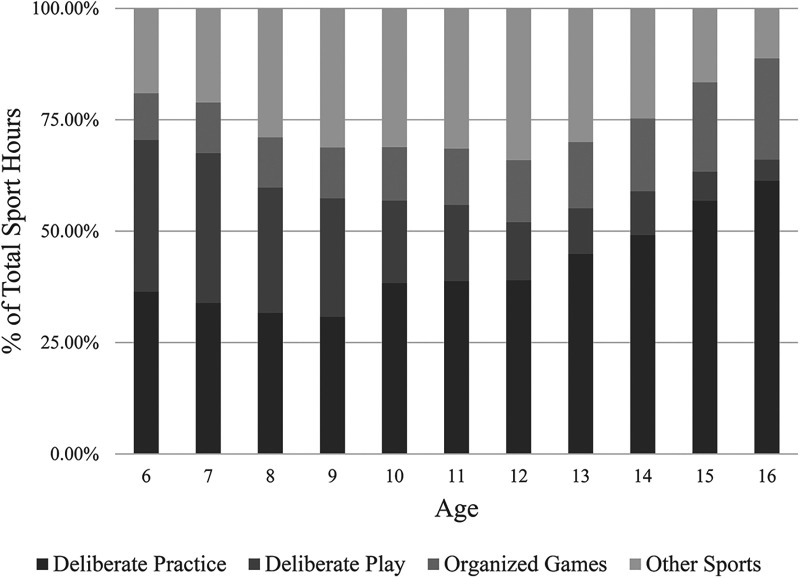
Percentage of total sport hours divided by hockey-specific deliberate practice activities, hockey-specific deliberate play, organized hockey games and sports other than hockey at each age.

To illustrate the proportion of time participants spent in each of the four sport categories, Figure 2 shows the percentage of time that participants were engaged in each activity for each year during the developmental period. The proportion of time that participants allocated to deliberate practice increased steadily from a low of 30.75% at 9 years of age to a high of 61.31% at 16 years. In contrast, the proportion of time that participants allocated to deliberate play decreased each year from the high of 34.06% at 6 years old to 4.71% at age 16. The proportion of time that participants allocated to organized hockey games showed small increases from 6 (10.48%) to 12 (13.93%) years of age, and then steadily increased from ages 13 (14.85%) to 16 (22.73%). At 12 years old, participants allocated the greatest proportion of time to other sports (34.08%), but the time committed to these activities decreased sharply to its lowest value (11.24%) at 16 years old.

### 3.3. Developmental milestones

In the second section of Côté et al.'s (64) interview script, athletes are asked questions related to 31 developmental milestones throughout their hockey careers. Participants in the current study indicated that they were, on average, approximately 3 years old when they first started playing hockey, and 4 years old when they became regularly involved in the sport (see Table 3). At an average of approximately 5 years of age, participants became involved in competition at the recreational level, and the majority were recognized among the best five players at that level. By 9 years old, most participants were playing competitive hockey, and most were recognized as being among the best five players by 10 years of age. Seven of 15 participants had competed at the national level, with five taking part in international competition. The idea of becoming an elite athlete emerged between 3 and 14 years of age, while the decision to become an elite athlete was made between 6 and 15 years of age. Most participants first engaged in regular team training by age 8 and started non-sport specific training by age 12. By the age of 13, most participants had completed their first off-season training camp, and by 14 years old, most acknowledged that they spent all their available leisure time training for hockey. When looking toward their athletic futures, participants thought they would reach their maximum athletic potential, on average, at approximately 23 years of age, and would retire from competitive hockey at an average age of 33 years.

**Table 3:** Descriptive statistics for first emergence of developmental milestones (in years).

Milestone question		Statistic					
		Mean	S.D.	Median	Mode	Max	Min
Regular involvement in hockey		3.87	0.74	4	4	5	3
Involvement in supervised training		5.47	1.81	5	4/5	10	3
Involvement in unsupervised training		8.13	3.23	8	4/7	14	4
Started playing hockey		3.40	0.74	3	3	5	2
Played in an organized league		4.80	0.94	5	4	7	4
Sport specific training regularly		12.33	2.35	13	14	15	7
Non-sport specific training regularly		12.07	2.02	12	12	15	8
Idea for becoming an elite athlete		9.53	4.21	8	14	14	3
Engages in the regular training of a team		8.47	3.76	8	4/12	14	4
Decision was made to become an elite athlete		11.73	2.69	12	14	15	6
All available leisure time was spent on training		12.93	2.74	14	14	16	7
Off-season training camp		12.87	2.13	13	14	16	9
Relocated for hockey		16.07	0.83	16	16/17	17	15
Established an extended relationship with a coach		13.14	2.48	12	12	18	9
You will reach your maximum potential		23.4	3.29	24	20/28	28	19
You will retire from competitive hockey		33.2	4.74	35	35	38	19
Competition level	*n*						
Competition at the recreational level							
Age for first participation	15	5.00	0.93	5	5	7	4
Recognized as a top 5 player	14	5.43	1.28	5	4/5	8	4
Recognized as the best player	14	6.50	2.14	6	6	12	4
Competition at the competitive level							
Age for first participation	15	9.00	2.33	9	7	13	6
Recognized as a top 5 player	15	9.87	2.70	10	12	14	6
Recognized as the best player	15	11.20	3.28	12	12/15	15	6
Competition at the all-star level							
Age for first participation	13	13.15	2.12	14	14/15	15	8
Recognized as a top 5 player	9	13.78	1.39	15	14	15	11
Recognized as the best player	3	13.67	1.53	14	12/14/15	15	12
Competition at the national level							
Age for first participation	7	15.57	0.79	16	16	16	14
Recognized as a top 5 player	5	15.40	0.89	16	16	16	14
Recognized as the best player	3	15.67	0.58	16	16	16	15
Competition at the international level							
Age for first participation	5	15.40	0.89	16	16	16	14
Recognized as a top 5 player	3	15.67	0.58	16	16	16	15
Recognized as the best player	2	15.50	0.71	15.50	15/16	16	15

Additional quantitative and qualitative information was obtained from the interviews regarding each participant's developmental history. This information was compiled and presented for each year of the survey. Of note, participants engaged in various levels of hockey at younger ages, but all athletes were playing at the highest level of youth hockey by the age of 12 (refer to Table 4). While five participants in the sample played against older players at the ages of 6 and 7, only two were doing so at 8 and 9 years of age, and only one remained in an older age group thereafter (see Table 5). Only one injury was reported before the age of 12, but a total of 22 injuries were reported between the ages of 12 and 16. Three of these injuries were considered chronic, or overuse, while the other 19 were acute in nature (Table 6). Fourteen years was the earliest age identified at which a participant first reported an injury affecting his current performance, despite being able to participate with the condition. This portion of the interview also involved the collection of subjective information regarding the perceived intensity of training, and the available resources at each age (see Table 7). Both steadily increased throughout participants' development from the lowest values at the age of 7, until attaining maximum values at 16 years old. Finally, this section of the interview asked participants to calculate the number of hours spent on hockey activities at different ages. At each age, players understated their involvement in hockey activities, compared to the following section when they analyzed each age more thoroughly.

**Table 4:** Number of participants at each level of hockey for each age.

Level	Number of participants at each age										
	6	7	8	9	10	11	12	13	14	15	16
House	4	3	1	0	0	0	0	0	0	0	0
Novice	1	2	1	0	0	0	0	0	0	0	0
Selects	2	2	0	0	0	0	0	0	0	0	0
Travel	1	2	2	2	1	0	0	0	0	0	0
Rep	1	1	1	1	1	1	0	0	0	0	0
A	1	3	3	3	3	0	0	0	0	0	0
AA	1	1	3	4	1	0	0	0	0	0	0
AAA	1	1	4	5	9	14	15	15	15	10	0
Academy	0	0	0	0	0	0	0	0	0	0	1
Junior C	0	0	0	0	0	0	0	0	0	0	1
Junior B	0	0	0	0	0	0	0	0	0	1	4
O.H.L.	0	0	0	0	0	0	0	0	0	4	9

O.H.L., Ontario Hockey League.

**Table 5:** Participants’ ages relative to that of other players in their respective leagues.

Age of other players	Number of participants at each age										
	6	7	8	9	10	11	12	13	14	15	16
Two years older	1	1	0	0	0	0	0	0	0	0	0
One year older	5	5	2	2	0	0	1	1	1	1	1
Same age	6	9	13	13	15	15	14	14	14	14	14

**Table 6:** Information regarding injuries for hockey related activities for each age.

Injury question	Age										
	6	7	8	9	10	11	12	13	14	15	16
Injury reported	0	0	0	1	1	0	4	4	3	3	8
Acute mechanism	NA	NA	NA	1	1	NA	3	2	3	3	8
Overuse mechanism	NA	NA	NA	0	0	NA	1	2	0	0	0
Combined time away	NA	NA	NA	30 d	30 d	NA	154 d	210 d	111 d	111 d	328 d
Combined affected time	NA	NA	NA	30 d	30 d	NA	288 d	285 d	5 yr	5 yr	5 yr

d, days; yr, years.

**Table 7:** Descriptive statistics for intensity, resources, hockey activity hours reported, and hockey activities calculated.

Category	Age										
	6	7	8	9	10	11	12	13	14	15	16
Intensity (subjective ratings on a scale of 0–100)											
Mean	53.33	51.33	53.67	54.67	61.33	67.00	70.67	76.00	78.33	82.67	96.33
S.D.	25.35	26.69	27.42	27.80	23.34	16.78	16.99	13.12	14.72	16.78	6.67
Median	50	50	60	60	60	65	65	75	80	85	100
Max	100	100	100	100	100	100	100	100	100	100	100
Min	10	10	10	10	20	40	50	60	60	60	80
Resources (subjective ratings on a scale of 0–100)											
Mean	59.17	57.33	58.67	61.33	65.33	71.33	74.33	78.33	78.67	85.33	93.00
S.D.	26.78	26.04	26.69	24.46	22.00	17.37	18.11	16.55	16.53	14.20	9.96
Median	55	50	60	60	60	70	70	80	80	85	100
Max	100	100	100	100	100	100	100	100	100	100	100
Min	10	10	10	30	30	50	50	50	50	50	70
Hours reported											
Mean	157.67	154.13	185.60	196.80	258.40	288.27	327.20	388.00	405.87	616.27	832.00
S.D.	90.23	77.90	115.63	112.65	175.60	164.98	190.10	170.47	198.03	443.92	365.97
Median	126	120	140	140	192	240	256	336	336	476	800
Max	336	336	480	480	660	660	768	768	880	1,920	1,920
Min	48	72	96	96	96	88	88	144	192	192	336
Hours calculated											
Mean	614.00	711.33	772.93	786.53	913.87	951.20	990.93	1,077.20	1,065.33	1,062.13	1,183.20
S.D.	521.28	499.27	491.50	484.02	697.86	706.38	684.20	712.35	616.49	514.10	496.65
Median	504	504	612	612	660	660	756	824	824	868	980
Max	1,792	1,792	1,816	1,816	2,544	2,544	2,544	2,544	2,356	2,208	2,716
Min	0	184	192	312	312	312	344	344	416	416	736

### 3.4. Effort, concentration, and fun

In addition to calculating the number of hours in each of the hockey activity categories, participants were asked to subjectively rate the “physical effort”, “mental concentration”, and “fun” for each hockey activity that they identified throughout their development. Table 8 outlines the descriptive statistics of the responses for organized games. The median value of the participants' responses regarding physical effort increased steadily from 6 years of age (82.5%) to 16 years (100%), with the exception of when the participants were 10 years old, which decreased slightly (81.67%) from when they were 9 years of age (85%). The reported “mental concentration” of participants followed a similar trend, with increasing median values from ages 6 (85%) through 16 (100%). “Organized games” were seen as enjoyable by the participants from 6 to 15 years of age, with a median “fun” rating of 100% during this timeframe. However, the median (80%) decreased considerably once participants turned 16 years of age. Similarly, participants rated “practices” (see Table 9) with increasingly higher levels of “physical effort” and “mental concentration” from ages 6 until 16, with both having maximum (100%) median ratings at 16 years of age. Much like organized games, the median of reported ratings regarding “fun” for practices was 100% from 6 years old until the median decreased substantially to 75% at 16 years of age. Finally, participants rated self-initiated activities (summary of results available by request) as relatively constant throughout their development, with the “physical effort” median ratings reported as being from 80% to 90%. The “mental concentration” reported by participants had a median rating of 70% until the age of 10, when it increased steadily to a median of 95% at 16 years old. Lastly, self-initiated “fun” ratings were highest early in participants' developmental histories, with a median of 100% at 6 years old. The reported “fun” ratings then decreased to a median of 85% at 15 years old and remained the same at 16.

**Table 8:** Subjective ratings of effort, concentration, and fun for organized games (subjective ratings on a scale of 0–100).

Category	Age										
	6	7	8	9	10	11	12	13	14	15	16
Physical effort											
Mean	80.00	81.33	82.00	83.67	81.67	85.53	88.87	91.53	91.67	94.00	98.00
S.D.	22.26	22.56	19.62	20.22	19.97	18.69	14.07	11.81	11.90	11.83	5.61
Median	82.5	85	85	90	85	98	98	98	100	100	100
Max	100	100	100	100	100	100	100	100	100	100	100
Min	40	40	40	40	40	40	60	60	60	60	80
Mental concentration											
Mean	71.67	76.00	76.67	78.00	78.00	81.33	84.33	88.33	89.67	93.33	97.33
S.D.	24.80	22.61	18.39	19.35	20.07	16.85	14.00	11.90	12.02	11.75	7.99
Median	85	80	80	80	80	80	80	90	90	100	100
Max	100	100	100	100	100	100	100	100	100	100	100
Min	40	40	50	50	50	50	60	60	60	60	70
Fun											
Mean	97.50	97.00	97.67	97.67	94.33	98.00	97.00	95.67	94.33	90.00	81.67
S.D.	5.00	7.97	7.76	7.76	14.50	7.75	8.41	8.63	9.42	14.14	18.29
Median	100	100	100	100	100	100	100	100	100	100	80
Max	100	100	100	100	100	100	100	100	100	100	100
Min	85	70	70	70	50	70	70	70	70	60	50

**Table 9:** Subjective ratings of effort, concentration, and fun for practices (subjective ratings on a scale of 0–100).

Category	Age										
	6	7	8	9	10	11	12	13	14	15	16
Physical effort											
Mean	78.33	78.67	75.67	77.33	79.67	80.33	84.33	87.67	88.33	91.67	96.67
S.D.	21.78	22.40	21.78	22.19	22.40	21.25	17.82	15.68	16.00	16.00	10.47
Median	77.5	80	80	80	80	80	90	90	90	100	100
Max	100	100	100	100	100	100	100	100	100	100	100
Min	40	40	40	40	40	40	40	40	40	40	60
Mental concentration											
Mean	61.25	63.33	62.33	64.00	71.33	72.67	80.00	83.33	84.00	88.33	93.00
S.D.	29.70	30.10	29.57	30.89	28.50	28.65	18.90	17.59	17.24	18.09	13.34
Median	65	70	60	60	80	80	80	80	80	90	100
Max	100	100	100	100	100	100	100	100	100	100	100
Min	0	0	0	0	0	0	30	30	30	30	50
Fun											
Mean	97.50	93.00	92.33	92.33	95.00	96.00	95.33	92.33	91.00	88.67	77.33
S.D.	5.84	16.01	15.91	15.91	13.23	12.98	13.02	13.48	13.65	15.17	19.35
Median	100	100	100	100	100	100	100	100	100	100	75
Max	100	100	100	100	100	100	100	100	100	100	100
Min	85	50	50	50	50	50	50	50	50	50	50

### 3.5. Other sports

Information regarding the other sports that the participants engaged in while growing up was compiled and categorized by sport type (available in Table 10). At 10 years old, all participants involved in this study played at least one invasion sport other than hockey, and at 12 years old, 14 of the participants amassed the greatest combined total hours (1,942), mean hours (129.47), and median hours (108) in invasion sports. At 15 years old, participation in invasion sports dropped substantially, with only six athletes accumulating any hours whatsoever. Eleven participants engaged in run scoring/field sports at the ages of 10 and 11, but the greatest combined total of hours from all participants came at 12 years old (800). By 14 years of age, only a minority of participants were involved in run scoring/field sports, and there were only two athletes participating in these sports by the age of 16. Net/wall sports had the highest combined total hours (1,110), maximum hours for a participant (528), and number of participants (12) at age 13, but by age 15 less than half of the participants reported any involvement. Participation in target sports followed a different trend as the highest combined total hours (984), median (32), and maximum number of participants (11) occurred at the age of 16. The highest number of participants in individual sports (i.e., 10) occurred between the ages of 10 and 13, and only six participants reported any involvement by the age of 14. Additionally, at 12 years old, participants accumulated the highest combined number of hours in individual sports (528). Invasion sports accrued the most hours at each age until 15 years, when target sports became the most prominent, according to the total number of hours accumulated. The gain in the relative popularity of net/wall sports is second only to invasion sports at age 13 and were the second most popular type of sport at the ages of 15 and 16.

**Table 10:** Descriptive statistics for hours of sports by type and age.

	Age										
	6	7	8	9	10	11	12	13	14	15	16
Invasion sports (excluding hockey)											
Total hours	632	822	998	1,262	1,498	1,586	1,942	1,476	1,074	382	198
Mean hours	42.13	54.80	66.53	84.13	99.87	105.73	129.47	98.40	71.60	25.47	13.20
S.D.	48.1	54.4	79.8	96.9	89.9	93.7	112.4	102.3	62.1	45.5	27.2
% sport hours	8.57	9.23	9.47	11.37	12.21	11.87	13.13	9.26	7.11	2.74	1.31
Median	32	32	40	40	96	96	108	80	72	0	0
Max	168	168	288	384	384	384	488	392	168	168	96
Min	0	0	0	0	16	0	0	0	0	0	0
Athletes (#)	11	12	11	14	15	14	14	14	11	6	4
Run scoring/field sports											
Total hours	348	420	644	704	692	772	800	700	560	240	40
Mean hours	23.20	28.00	42.93	46.93	46.13	51.47	53.33	46.67	37.33	16.00	2.67
S.D.	51	50	61	61	52	70	73	58	59	36	7
% sport hours	4.72	4.72	6.11	6.34	5.64	5.78	5.41	4.39	3.71	1.72	0.26
Median	0	24	24	32	36	36	32	24	0	0	0
Max	200	200	200	200	200	280	280	200	200	120	24
Min	0	0	0	0	0	0	0	0	0	0	0
Athletes (#)	6	8	10	10	11	11	10	9	7	4	2
Net/wall sports											
Total hours	0	80	80	168	320	500	710	1,110	580	412	216
Mean hours	0.00	5.33	5.33	11.20	21.33	33.33	47.33	74.00	38.67	27.47	14.40
S.D.	0	21	21	37	41	54	55	130	71	54	29
% sport hours	0.00	0.90	0.76	1.51	2.61	3.74	4.80	6.97	3.84	2.95	1.42
Median	0	0	0	0	0	24	32	32	12	0	0
Max	0	80	80	144	144	208	208	528	272	208	96
Min	0	0	0	0	0	0	0	0	0	0	0
Athletes (#)	0	1	1	2	5	8	11	12	8	7	4
Target sports											
Total hours	176	208	448	416	452	484	696	696	712	808	984
Mean hours	11.73	13.87	29.87	27.73	30.13	32.27	46.40	46.40	47.47	53.87	65.60
S.D.	28	28	79	76	76	77	79	79	78	79	84
% sport hours	2.39	2.34	4.25	3.75	3.68	3.62	4.71	4.37	4.71	5.79	6.49
Median	0	0	0	0	0	0	12	12	32	32	32
Max	96	96	300	300	300	300	300	300	300	300	300
Min	0	0	0	0	0	0	0	0	0	0	0
Athletes (#)	4	5	5	6	7	7	9	9	10	10	11
Individual sports											
Total hours	240	236	400	384	476	500	528	504	336	228	116
Mean hours	16.00	15.73	26.67	25.60	31.73	33.33	35.20	33.60	22.40	15.20	7.73
S.D.	24	24	32	33	31	34	36	33	32	27	19
% sport hours	3.25	2.65	3.80	3.46	3.88	3.74	3.57	3.16	2.22	1.63	0.76
Median	8	0	16	16	24	24	24	24	0	0	0
Max	80	80	96	96	80	104	104	84	84	80	72
Min	0	0	0	0	0	0	0	0	0	0	0
Athletes (#)	8	7	9	8	10	10	10	10	6	5	3

Percentage of sport hours for each type/category calculated with hockey included.

Table 11 outlines specific sport involvement. Soccer was played by all the athletes that were interviewed at some point during their development. From the ages of 6 through 12, at least ten athletes participated in soccer; however, only one participant was involved in soccer at the age of 15. Baseball, basketball, golf, and volleyball shared the second most popular spot, with 12 participants taking part at some point during their development. Golf was popular among five participants at 6 years of age and reached its maximum of 12 participants at 15 years old. At 12 years, 5 sports (i.e., soccer, baseball, basketball, golf, and volleyball) had ten or more participants, while at 14 years of age, only golf had more than seven participants. By the age of 15, no sport other than golf had more than four participants.

**Table 11:** Specific sport participation by age.

Sport	Number of participants at each age											Total
	6	7	8	9	10	11	12	13	14	15	16	
Soccer	11	11	10	10	11	11	11	7	6	1	1	15
Baseball	7	9	9	10	10	11	10	9	7	3	2	12
Basketball	2	2	3	7	8	9	11	12	6	2	0	12
Golf	5	6	6	7	8	8	11	11	12	12	11	12
Volleyball	0	0	0	2	4	7	10	11	6	3	2	12
Athletics	2	2	5	5	6	6	7	7	4	3	1	9
Badminton	0	7	0	0	2	4	7	7	3	1	1	7
Lacrosse	0	0	1	5	6	4	4	3	2	0	0	6
Swimming	5	6	6	5	4	3	2	2	2	2	2	6
Football	0	1	3	3	3	4	4	4	4	4	4	5
Cross country	1	1	1	1	4	3	3	2	2	0	0	4
Tennis	0	0	0	0	0	1	2	2	2	2	2	3
Gymnastics	2	0	0	0	0	0	0	0	0	0	0	2
Dodgeball	0	1	1	1	1	1	1	1	1	1	1	1
Incline hockey	0	1	1	1	1	1	1	0	0	0	0	1
Ultimate frisbee	0	0	0	1	1	1	1	1	1	0	0	1

### 3.6. Supplemental questions related to specialization

To supplement the Côté et al. (64) interview guide, several questions were asked to identify other aspects of sport specialization. Three of these questions were proposed by Jayanthi et al. (62), which helped assign a degree of specialization to participants based on their responses. Participants in this study stated that they chose “hockey as their main sport” by 11.67 years of age, on average, with a median of 13 years old. The mean for “quitting other activities to play hockey” was 12.47, with a median of 12 years old. Finally, participants responded that they “trained for hockey for more than eight months a year” at 12.40 years of age, on average, with a median of 13 years old.

Due to participants reaching each criterion at different ages, the responses from individual participants were analyzed to determine the combined degree of specialization. The reported answers indicate that the participants in this study were “moderately specialized” in hockey at a mean of 12.33, and a median of 13 years of age, and “highly specialized” at a mean of 13.40, and a median of 14 years. However, upon calculating the duration of annual training via answers to the “hockey activity” portion of the interview, it appears that participants underestimated their annual commitment to hockey. Based on these calculations, the mean age at which training occurred for more than eight months in a given year was 7.8 years, with a median of 7 years. These calculations indicate that the mean age at which the participants became highly specialized in hockey was slightly younger at 12.87 years of age (vs. reported mean age of 13.40 years), although the median age remains at 14 years (see Table 12).

**Table 12:** Answers to questions regarding the degree of specialization (age in years).

	Statistic					
	Mean	S.D.	Median	Mode	Max	Min
Specialization questions						
Consider hockey your main sport?	11.67	3.39	13	14	15	5
Quit other activities to play hockey?	12.47	1.85	12	12	15	8
Train for hockey more than 8 months a year?	12.40	2.92	13	13/14/15	16	7
Calculated training for more than 8 months	7.80	2.46	7	6	14	6
Reported specialization degree						
Moderately specialized	12.33	2.53	13	14	15	7
Highly specialized	13.40	1.76	14	15	16	10
Calculated specialization degree						
Moderately specialized	11.00	3.09	12	13	15	6
Highly specialized	12.87	1.88	14	14	15	8

Finally, four questions regarding issues of specialization were asked of each participant. Eight participants were instructed to not participate in other sports during the hockey season at some point during their development. In five of these cases, coaches made the requests independently, while both coaches and parents made the requests in the remaining cases. In each instance, participants were prevented from participating in other sports to prevent potential injuries. Only two participants were instructed not to play other sports during the off-season, and both the parents and coaches made these requests, again, to prevent injuries from occurring. Only one participant had ever attended a hockey academy, and he attended this academy for a total of one year. Of the 15 athletes interviewed for the study, 11 had played for more than one minor hockey association, with nine participants having relocated to play against higher levels of competition; one to avoid conflict with a coach and one was cut from his previous team.

## 4. Discussion

The purpose of this study was to describe and analyze the developmental histories of a group of current and former OHL players. Thus, the results concentrate on the years prior to participants’ involvement in this league, which may commence as young as 16 years old, with the rare instance of “exceptional status” being granted to players who are 15 years old (75). The median of 139 OHL games played shows that the majority of participants had been involved in more than two complete seasons in the League at the time of their interviews. These participants created a homogeneous sample of skilled hockey players by having maintained OHL status, thereby showing a high level of proficiency, which is a common determinant of expert status (76). As hypothesized, participants reported involvement in hockey competition prior to the age suggested by Hockey Canada and showed high levels of commitment to hockey as young as 9 years old. However, the participants' backgrounds also featured a diverse sampling stage early in their athletic careers.

### 4.1. Hockey-specific deliberate play

Collectively, participants of the current study spent approximately 22% of their total sports hours in the sampling stage (age 6–13) engaged in deliberate play activities and only 8% during the specialization stage (age 13–15). A large decrease in the combined total of deliberate play hours was observed between the ages of 9 and 10, as well as between 14 and 15. While hockey-specific comparisons are limited in the literature, Soberlak (77)^7^ reported 55% and 30% of hours spent in deliberate play activities during sampling and specialization stages, respectively for CHL players (*n* = 4). This may suggest that deliberate play hours were on the lower end in this particular sample of OHL players. Using the same criteria as Soberlak and Côté (25), participants of the current study may have been considered to be specialized as early as 10 years old and invested by 15 years of age. These ages are younger than those suggested by both Hockey Canada (43) and the DMSP (41).

The reduction of sport-specific play activities noted in this study may negatively affect some elements of development that assist with improving performance. Roca and colleagues (78) found that 21.8% of the variance found on a soccer decision-making task was accounted for by participants' accumulated childhood soccer-specific play hours. Moreover, Ford et al. (79) found that soccer-specific play was the only developmental activity that differentiated professionals from less successful players. In studies of sport-specific creativity, hours spent in unstructured play differentiated the “most creative” athletes from the “least creative” within team ball sports (80). An 18-week deliberate-play, basketball intervention suggested similar benefits with respect to tactical creativity and tactical intelligence (81). Free play has also been tied to the development of intrinsic motivation for the activity (82, 83); although this view is not universally accepted [e.g., (84)].

### 4.2. Hockey-specific deliberate practice

While the difference between practice and play may not always be clear (85), deliberate practice in the current study included any activity (e.g., self-initiated practice, team practice, individual coaching) that was done to improve performance (64). The increase in accumulated hours of deliberate practice reported from 9 to 10 years of age may be explained by the increase in the number of players competing at the highest level of youth hockey (i.e., AAA). The difference in accumulated hours of deliberate practice between 12 and 13 years old may be explained by the advancement of participants from the “peewee” to “bantam” age categories^8^. Similarly, between the ages of 15 and 16, participants were transitioning to the OHL, where time commitments to the sport are much greater. Corresponding with this increased investment was the reduction of fun reported by participants, such that by the age of 16 hockey was not as enjoyable as in previous years. For comparison purposes, only 10% of the hours collected by the junior hockey players in the Soberlak and Côté (25) study were dedicated to deliberate practice in the sampling stage (age 6–13), whereas 35% of all sports hours were spent on deliberate practice in the current sample. Deliberate practice during the specializing stage (age 13–15) accounted for 18% of the Soberlak and Côté (25) participants' sport hours, and 50% of the hours reported by the participants of this study.

Participants appeared to underestimate their commitment to hockey when asked to summarize the time spent in hockey-related activities. When the total number of hours spent in activities specific to hockey were calculated manually by the research team, there were more than double the number of hours reported. While the interview script used in this study allowed for the distinction between deliberate play and deliberate practice, Côté et al. (85) suggest that a continuum exists between the two forms of developmental activities. Thus, the difference between the two activities might not be easily recognized. The general trend of participants' increasing amounts of deliberate practice at the expense of play activities found in this study is consistent with previous research (86, 87). However, while some studies have found that higher amounts of accumulated sport-specific practice results in better performance [e.g., (72, 76)], others find that expert status is not explained by accumulated practice hours alone [e.g., (88, 89)]. One explanation for the early accumulation of deliberate practice hours in youth sports is that some coaches pressure young athletes into training at higher levels than needed due to an overemphasis on winning (90). Valuing winning at the expense of player development creates an adult-centric environment that rationalizes specialization (91).

### 4.3. Organized hockey games

The hours that participants in the current study spent competing in organized hockey games increased throughout their developmental years in similar proportions to that found by Soberlak and Côté (25). This may suggest that the proportion of time spent competing in organized games has not changed substantially for athletes of this level since Hockey Canada implemented changes to its grassroots programs after the Open Ice Summit. However, the majority of participants in the current study started playing hockey by the time they were 3 years old, began regular involvement by 4 years old, and were competing in an organized league when they were 5 years of age. This is considerably younger than the hockey players in the Wall and Côté (92) study where, on average, participants started playing after they were 5 years old, and initiated competition when they were 6 years of age. The younger ages found in the current study suggest that Canadian hockey players are starting to engage in organized hockey at younger ages than has been documented in previous research, although further investigations are warranted to draw conclusions.

### 4.4. Other sports

The total number of accumulated hours that participants played sports other than hockey increased until the age of 12 and then decreased every year thereafter. The number of sports played other than hockey increased from an average of 2.0 at the age of 6, to a maximum average of 5.6 at 12 years old, and then decreased each year to 2.3 at 15 years old. Comparatively, Soberlak (77) found that the junior hockey players in his study played a similar number of sports (on average, 3 sports between ages 6–8 and 6 sports between 9 and 12) but did not decrease the amount of time spent playing other sports until the age of 14 (25). Further, a lower proportion of the sampling stage was spent participating in sports other than hockey relative to the specialization stage (77). This suggests participants in the current study may have spent more time playing sports other than hockey early in their development, but ceased playing these other sports sooner than previous research on junior ice hockey players has indicated. However, the participants of the current study began to eliminate sports other than hockey at an age that is consistent with Hockey Canada's (43) LTAD program, and the DMSP's (41) recommendations regarding participating in other sports.

Consistent with findings in this study, previous work has shown that expert athletes in other sports tend to participate in several sports until the age of 12, at which point they narrow their focus [i.e., (66, 91, 93)]. Likewise, athletes who played a variety of sports at the age of 12 have better results in several measures of athletic performance (94). Conversely, Ford et al. (79) found no difference in the number of hours spent playing other sports between elite and non-elite soccer players. While the authors did not find an advantage in playing different sports early in one's development, they also found that doing so did not hinder the chances of attaining elite status.

In addition to estimating the number of hours played in sport other than hockey, the type and classification of sport was also collected. The 15 participants in the current study indicated that they competed in a combined 16 sports, other than hockey, from all five sport categories, displaying the diverse developmental pathways noted by other authors [e.g., (95)]. While the extent to which skills may transfer across sports is not universally accepted, there is evidence to suggest that similar sports have elements of positive skill or tactical transfer [e.g., (91, 96–98)]. Consistent with previous research, the number of hours that participants of the current study engaged in invasion sports is greater than the hours accumulated in other categories (72). The similarity of tactics employed in invasion sports may increase the likelihood for transfer because athletes can “chunk” information, allowing them to broaden their focus and process more stimuli (96, 98, 99). Therefore, participants may have enhanced hockey performance due to their participation in these sports by a mechanism known as lateral, or near, transfer (100), where the training outcomes in one sport can be used in other sports (101).

### 4.5. Supplemental questions related to specialization

In the current study, most participants considered hockey their main sport by the age of 13, quit other activities for hockey by the age of 12, and reported training more than eight months per year for hockey at 13 years old. Due to athletes reaching each criterion at different ages, the degree of specialization was calculated individually, and then analyzed. Accordingly, most of the participants in the current study were considered “moderately specialized” at 13 years old, and “highly specialized” at 14 years of age (based on median values). Using the detailed information derived from the Côté et al. (64) interview script, the age at which participants in the current study trained for eight or more months per year could be empirically calculated. Based on these calculations, most participants in the current study had trained for eight or more months per year by 7 years of age, as opposed to their general estimation of 13 years old. Moreover, the calculated age at which participants trained for more than eight months per year indicates that they were moderately specialized at 12 years of age, which is one year earlier than the 13-year-old average the participants reported in their responses to the supplemental questions. In reference to the age divisions for minor hockey in Canada, this one-year disparity is the difference between a player competing at the “bantam” vs. “peewee” level (102). The calculated median age of participants being categorized as highly specialized was 14 years, which is consistent with the average reported in their answers to the supplemental questions. However, most of the participants answered that they had the idea to become an elite athlete by 8 years old and decided to become one by 12 years of age. These qualitative indications suggest that participants may have been highly specialized prior to 14 years of age.

Research has shown that being classified as highly specialized is an independent risk factor for reporting an injury, regardless of training volume and age (50, 62, 103). Jayanthi and colleagues (62) also stated that athletes who exceed a 2:1 ratio of organized training (i.e., deliberate practice and organized games) to free play (i.e., deliberate play) were more likely to develop an injury. Accordingly, this ratio was achieved by most of the participants of the current study by 12 years of age, which corresponds to when the first participant reported playing through an injury. Empirically calculating the age at which these participants became highly specialized may provide insight into the injuries that they reported starting in that year.

It has been reported that some youth coaches prohibit athletes from participating in sports other than their primary sport (12). In all eight instances of participants in this study being instructed to not play other sports, the rationale was to prevent injuries that may inhibit their performance in hockey. However, the recommendation may be ill-advised as specialization has been closely associated with overuse injuries, and athletes may suffer acute injuries at any time in their main sport as well (62, 93, 104). Youth sport organizations also place pressure on parents and coaches to promote early specialization by placing value in the result of competition, and devaluing the developmental process (91). Pressure is placed on coaches to win by these organizations by advertising national rankings as young as 4 years old (105, 106). Likewise, parents feel pressured to push specialization as third-party entities attempt to forecast their children's professional outlook as early as 7 years old (105).

Only one of the participants in the current study attended a hockey academy, and only for a single year. This participant attended an academy at the age of 16, prior to attaining his position on an OHL team. Despite the proliferation of hockey academies throughout Canada, and specifically Ontario (60), they did not appear to have a major influence on the development of the OHL players in the current study. While hockey academies are becoming increasingly popular among minor hockey players, the four academies located within the region selected for the inclusion criteria did not begin operating until approximately 2014 (60, 107, 108). However, all participants in the current study reported using private instructors, such as skating and goalie coaches, as an element of their deliberate practice. Participants chose instructors for these sessions based on the specific skill(s) they were trying to enhance. Most participants in the current study began utilizing the services of individual coaches by 10 years old, while four participants started as early as 6 years of age. This means that the participants’ parents or guardians were responsible for paying for up to 10 years of individual coaching in an attempt to excel above their peers.

### 4.6. Strengths/limitations and future directions

The retrospective recall methodology used in this study provided invaluable insight into the developmental histories of these participants. However, most participants began hockey activities by 3 years of age and started competition at 5 years of age. The interview script prescribed that information be collected beginning at the age of 6 years; thus, future research examining the developmental histories of elite Canadian junior hockey players should account for the hours collected prior to 6 years of age to give a more accurate portrayal of athletes' sport involvement. Additionally, the retrospective answers that participants gave to the questions in Jayanthi et al.'s (62) three-point scale did not always substantiate the answers to similar questions in the Côté et al. (64) script. While the scale has demonstrated to be a valid tool in the study of specialization, it may not provide reliable information when used retrospectively. Future examinations of participant histories should aim to expand on these limitations, along with inclusion of larger samples to athletes (i.e., a notable limitation of previous work and the current study). Additional detailed analyses using alternative developmental models would also be beneficial. Rees et al. (109) concluded that both early specialization and early diversification (i.e., sampling and play) can lead to athletic success. Thus, the DMSP and LTAD models may have inherent limitations that require further exploration. Finally, while the Côté et al.'s (64) interview methodology is believed to provide valid and reliable information, participant memory recall is a potential limitation of retrospective studies of this nature.

Notably, participants consistently reported having enjoyed all their hockey pursuits despite an increase in structured activities. The fun that they experienced at a young age presumably allowed them to commit to the high levels of physical effort and concentration needed to develop into elite junior hockey players. Parents of children who aspire to play hockey at a high level should be aware that these participants consistently played sports other than hockey, and enjoyed their time spent in hockey activities. Therefore, finding ways to promote fun at a young age, regardless of the type of activity, appears to be instrumental in the development of elite junior hockey players.

### 4.7. Conclusion

Despite recommendations for delayed sport specialization among children, the early specialization path remains a popular trajectory among coaches, parents, and athletes alike in their pursuit of extrinsic rewards. This study provides detailed developmental histories of a group of elite junior hockey players from the OHL. The findings suggest the developmental pathways of elite junior hockey players may have changed since Hockey Canada altered its grassroots programs. However, despite diverse sampling activities early in their athletic careers, athletes reported evidence of specialization in hockey at young ages.

## Data Availability

The datasets presented in this article are not available on ethical grounds as they contain personal information.

## Footnotes

^1^From the early stages, a variety of sports have been studied to ascertain the relevance of various developmental activities, further reinforcing the interest and cultural importance placed on athlete achievement. Examples include tennis (20), field hockey (21), golf (22), soccer (23), volleyball (24), ice hockey (25), cricket (26), and gymnastics (27).

^2^Femoroacetabular impingement is characterized by ‘cam’ and ‘pincer’ bony abnormalities to the femoral neck or acetabular rim respectively (52).

^3^A copy of the interview script may be obtained from the corresponding author.

^4^‘Moderately specialized’ at a given age is indicated by two positive responses to any of the three questions. ‘Highly specialized’ is assigned for affirmative responses to all three questions at a particular age. Refer to Jayanthi et al. (62) for further discussion.

^5^‘Sport’ refers to all sports, including hockey.

^6^Examples of ‘artistic’ activities include music, dance, and drawing.

^7^Soberlak and Côté (25) refers to the published work that was derived from the more detailed Soberlak (77) Master's thesis. Both are referred to in the current manuscript, depending on where the information was provided.

^8^Hockey Canada modified the names used to identify developmental age groups for the 2022-2023 season. ‘Peewee’ and ‘bantam’ are now referred to as ‘U13’ and ‘U15’, respectively. This change occurred after the current participants progressed through the stages of development; thus, the former names are used throughout the manuscript.
